# Tasting evolution in a sea asparagus salad: Dissecting polyploid evolution and aquaporin function in *Salicornia*

**DOI:** 10.1093/plphys/kiaf636

**Published:** 2025-12-05

**Authors:** Guannan Wang

**Affiliations:** Assistant Features Editor, Plant Physiology, American Society of Plant Biologists; Department of Biology, Stanford University, Stanford, CA, USA; Howard Hughes Medical Institute, Stanford University, Stanford, CA, USA

Soil salinity poses an increasing threat to plant growth and development as climate change continues to intensify soil evaporation, seawater intrusion, and irregular precipitation patterns. The Food and Agriculture Organization of the United Nations (FAO) has recently estimated that over 10% of the total global land is already impacted by salinization, and the affected area may increase up to 32% by the end of the 21st century (FAO, 2024). Salt-induced soil degradation results in a global annual economic loss of US $27.3 billion (Qadir et al. 2014) and has become one of the major threats to agriculture and food security worldwide. Developing strategies to design salt-tolerant crops is crucial for reclaiming salt-affected land and meeting the challenge to feed the projected global population of 9.3 billion people by 2050 (FAO, 2024).

Halophytes, which are plants that have adapted to thrive in saline environments, represent a valuable yet underexplored reservoir for evolutionarily tested salt tolerance strategies. Because the trait of salt tolerance has likely evolved independently in multiple plant lineages, halophytes are widely distributed across flowering plant families, accounting for 1% to 2% of angiosperm species (Moray et al. 2015). A better understanding of adaptive diversity among halophytes will not only shed light on the evolutionary processes underlying plant adaptations to salinity stress but will also provide a catalog of strategies for designing climate-smart crops in changing environments. In the last decade, growing attention has been directed toward Amaranthaceae for halophyte studies as the largest number of halophytes are found in this family (Santos et al. 2016). Noteworthy among them are species from *Salicornia*, like *Salicornia europaea*, which is often found in coastal and inland salt marshes worldwide (Fig. A). These plants can tolerate more than 1,000 mM NaCl and have been considered as one of the most salt-tolerant plant groups (Jordine et al. 2024). While species like *S. europaea* serve as promising halophytic models for investigating salt-tolerance mechanisms, research on *Salicornia* has been considerably hindered by the genus's taxonomic complexity and unresolved polyploidy mystery. In this issue of *Plant Physiology*, Duan and colleagues (Duan et al. 2025) constructed a gap-free telomere-to-telomere genome assembly for tetraploid *Salicornia europaea*, investigated the polyploidization history of *Salicornia* species, and examined the role of aquaporins in regulating stress tolerance in this species.

**Figure.**
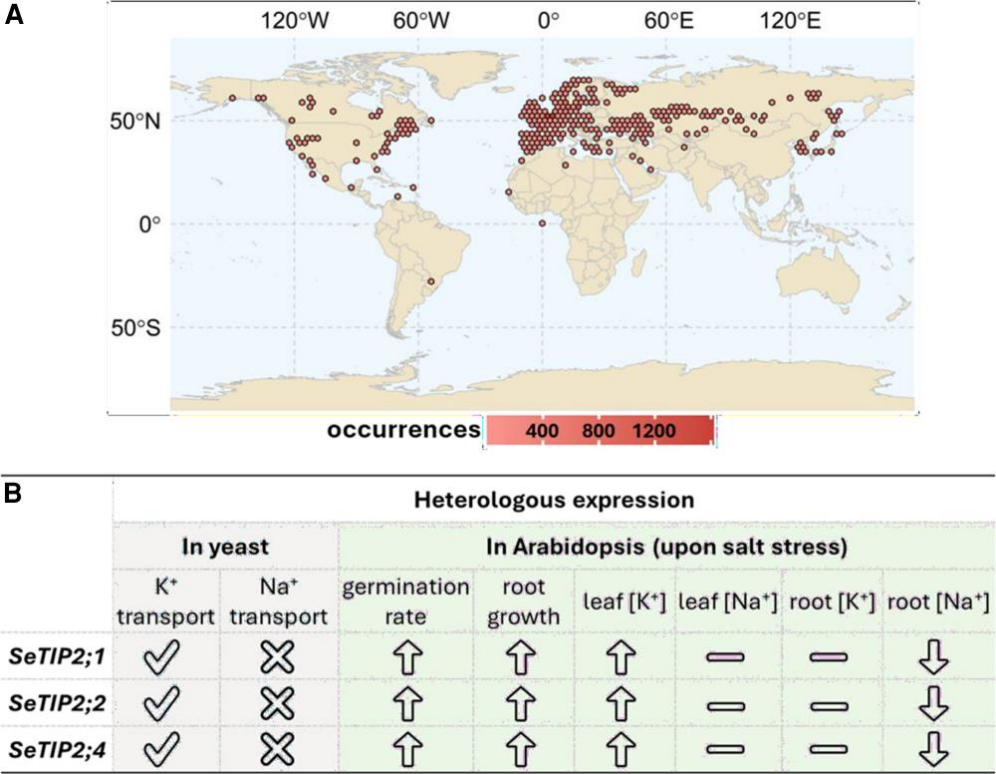
Global distribution and functional insights into *S. europaea*. **A)** Global distribution of *S. europaea* based on Global Biodiversity Information Facility (GBIF) database (GBIF.org^2025^). **B)** The effects of *SeTIP2;1*, *SeTIP2;2*, and *SeTIP2;4* on the growth of yeast and Arabidopsis.

The authors first assembled a draft genome for the tetraploid *S. europaea* using 2 major long-read sequencing technologies: Ultra-Long DNA sequencing from Nanopore and HiFi sequencing from PacBio, both of which produced reads that are longer than 10 kb. The draft genome was subsequently scaffolded using Hi-C sequencing data, which captures interactions between chromatins in 3-dimensional space. This generated a reference genome consisting of 18 pseudochromosomes with a total length of 984.58 Mb, similar to the estimate from flow cytometry. Using 3 different independent assembly measures that are often employed to assess the continuity and the content of newly assembled genomes, the authors confirmed that their reference genome is highly accurate and complete.

Two ancient whole-genome duplications were detected in *Salicornia* species prior to the emergence of the tetraploid *S. europaea* in contrast to 3 such events in *Suaeda glauca* from the same family. The 2 subgenomes in the tetraploid *S. europaea* exhibited both sequence and functional divergence, with subgenome A more closely resembling their diploid relative. Gene families exhibiting expansion or contraction in subgenomes A and B compared with ancestral *Salicorinia* were found to be involved in distinct biological processes. Diploid-contracted families were differentially retained between the subgenomes, with subgenome A exhibiting an expansion of more than 3 times as many such families as subgenome B. There were 19,453 structural variations (differences between genomes that are longer than 50 bp) detected between the tetraploid and diploid *S. europaea* but only 4,849 such variations between the subgenomes. Not surprisingly, the structural variations from different subgenomes or different polyploids are associated with distinct biological functions. These together suggest that the subgenomes in the tetraploid *S. europaea* have evolved differently.

Previous studies demonstrated that *Salicornia* plants, including *S. europaea*, can accumulate high levels of salt in both roots and shoots while avoiding ion toxicity (Lv et al. 2012). This high salt tolerance is achieved largely by compartmentalizing excess salt into the vacuoles to maintain stable cytoplasmic salt content. To better understand this subcellular compartmentalization, the authors investigated the expression of 16 predicted tonoplast-localized aquaporins (TIPs) out of the 53 aquaporins found in the tetraploid *S. europaea* genome. Among them, *SeTIP1;1/1;2*, *SeTIP2;1/2;3*, and *SeTIP2;2/2;4* were expressed at considerably higher levels in either root or shoot than other *SeTIPs* under both control and 100 mM-NaCl treatment conditions. Longer treatments with different salt concentrations further showed that *SeTIP2;1/2;3* and *SeTIP2;2/2;4* responded to the stress more dramatically compared with *SeTIP1;1/1;2*.

The authors subsequently cloned the coding sequences of *SeTIP2;1*, *SeTIP2;2*, and *SeTIP2;4* and demonstrated their vacuolar membrane localization in Arabidopsis protoplasts. Heterologous expression in yeast strain R5421, which lacks K^+^ transport activity, showed that both SeTIP2;1 and SeTIP2;2 could restore the growth of yeast on medium with low levels of K^+^, whereas SeTIP2;4 exhibited a markedly reduced ability to do so. However, none of the 3 TIPs were able to improve the growth of yeast strain AXT3K, deficient in plasma membrane Na^+^/H^+^ exchange activity, on medium with Na^+^, indicating that these SeTIPs might lack the capacity to transport Na^+^. The mechanism by which these SeTIPs can distinguish between the 2 highly similar monovalent cations, K^+^ and Na^+^, remains elusive.

The roles of *SeTIP2;1*, *SeTIP2;2*, and *SeTIP2;4* in stress tolerance were evaluated in Arabidopsis. Overexpression of these *SeTIPs* dramatically improved the germination rate and the root growth under salt stress (Fig. B). The lines overexpressing *SeTIP2;1* and *SeTIP2;4* also tend to exhibit higher leaf and root fresh weight and leaf water content upon salt treatment, albeit the variation observed across different plants. In root, K^+^ levels were largely indistinguishable between the wild type and overexpression plants, while Na^+^ concentrations were substantially reduced in the overexpression lines when they were exposed to salt stress. Conversely, in the leaf, the Na^+^ levels remained comparable across different lines, whereas K^+^ concentrations were significantly elevated in the overexpression lines (Fig. B). As a consequence, the overexpression plants maintained higher K^+^/Na^+^ ratios compared with the wild type, particularly in leaf. A similar pattern was also observed for soluble sugar content. The authors additionally assessed the contribution of these 3 *SeTIPs* to drought tolerance. Overexpressing these *SeTIPs*, especially *SeTIP2;4*, improved germination rate, primary root growth, leaf fresh weight, and leaf water content of drought-treated Arabidopsis. Taken together, these findings suggest that these *SeTIPs* likely contribute to plant adaptations to not only salinity stress but also drought stress.

In summary, the work by Duan and colleagues (Duan et al. 2025) presents a high-quality genome assembly for the tetraploid *S. europaea*, one of the most salt-tolerant plants, and reveals the functional divergence between the two subgenomes. Three *SeTIPs* from this species, *SeTIP2;1*, *SeTIP2;2*, and *SeTIP2;4*, have been demonstrated to regulate K^+^/Na^+^ homeostasis in plants and improve plant growth under both salt and drought stresses. This study raises several important questions for future research. For example, both the evolutionary trajectories of tetraploidization and the roles of subgenomes in stress adaptations remain unresolved.

Furthermore, it is unclear how the selected *SeTIPs* contribute to the differential distribution of salt between tissues and intracellular compartments and how they function synergistically with known Na**^+^** transporters, like HTK1 and SOS1, to enhance plant stress tolerance. Addressing these questions will not only help resolve the polyploidization mystery in *Salicornia* but will also provide a synergistic picture for how this species efficiently compartmentalizes excess salt—insights that could be leveraged to improve stress tolerance in stress-sensitive plants.

## Recent related articles in *Plant Physiology*:

- Zhao et al. (2024) investigated the development trajectory of salt glands in recretohalophyte *Limonium bicolor* using single-cell RNA-seq.
- Wang et al. (2023) revealed the role of alternative 3′-untranslated regions in stress adaptations in the halophyte *Spartina alterniflora*.

## Data Availability

No new data were generated or analysed in support of this research.
